# The Relation between Immunological Features and the Positive SARS-CoV-2 Nucleic Acid in Patients with Nonsevere COVID⁃19

**DOI:** 10.1155/2022/4270096

**Published:** 2022-07-30

**Authors:** Dong Zhang, Xueren Li, Haibai Sun, Lixia Shi, Shouchun Peng

**Affiliations:** ^1^Department of Tuberculosis, Haihe Hospital, Tianjin University, Tianjin 300350, China; ^2^Tianjin Institute of Respiratory Diseases, Tianjin 300350, China; ^3^Department of Respiratory Medicine, Haihe Clinical School of Tianjin Medical University, Tianjin 300350, China; ^4^Laboratory Medicine, Haihe Hospital, Tianjin University, Tianjin 300350, China

## Abstract

**Objective:**

The novel coronavirus nucleic acid results are the core indicators of illness monitoring. This study aimed to evaluate the relationship between immunological features and positive SARS-CoV-2 nucleic acid by analyzing the clinical and immunological features in nonsevere COVID-19 cases.

**Methods:**

Data from nonsevere COVID-19 patients admitted to Haihe Hospital from May 2020 to June 2021 were retrospectively reviewed and analyzed.

**Results:**

(1) A total of 122 cases were reviewed in the present study, including 38 mild and 84 moderate cases. The average age of mild cases was significantly different from moderate cases (*P* < 0.001). Eight patients complained of hyposmia and it was more frequent in mild cases (*P* < 0.001). The nucleic acid positive duration (NPD) of nonsevere novel coronavirus was 20.49 (confidence interval (CI) 17.50–3.49) days. (2) The levels of specific IgM and IgG for COVID-19 were higher in mild cases than in moderate cases (*P*=0.023 and *P*=0.047, respectively). (3) The correlation analysis with antibodies and T-cell subtypes showed that the lymphocyte (LYM) count, T cells, CD4^+^T cells, and CD8^+^T cells had a linear correlation with NPD. (4) Among the 93 patients monitored, 62 COVID-19 cases presented a progressive rise of specific IgM and IgG. The daily increase rates of IgM and IgG were 38.42% (CI 28.22–48.61%) and 24.90% (CI 0.23–29.58%), respectively.

**Conclusion:**

The levels and daily increase rates of specific IgM and IgG against the virus can vary between cases. The NPD presented a linear correlation with the LYM, T cells, CD4^+^T cells, and CD8+T cells. Hence, more attention should be paid to these indicators in clinical practice.

## 1. Introduction

The novel coronavirus disease 2019 (COVID-19) is an acute respiratory infectious disease that comprehends a serious risk to human health [1, 2] and has become a global pandemic. By November 1st, 2021, it has caused over 250 million infections and 5 million deaths worldwide [3]. At the molecular level, many parallels have been identified between SARS and COVID-19, and the COVID-19 virus has been named SARS-CoV-2 [4]. Many studies regarding COVID-19 have been performed and led to a certain understanding of its diagnosis, treatment, and prevention. COVID-19 patients can present different clinical symptoms including fever, fatigue, dry cough, myalgia, flu-like symptoms, and a very high mortality rate in severe and critical cases [5–7]. Some vaccines have been on the market and widely administered worldwide. However, there is still no effective drug to treat COVID-19. Hence, clinicians still rely only on the infected person's immune system to fight SARS-CoV-2.

The components of the human immune system, such as lymphocytes (LYM), and the subsets of CD4^+^ T cells, CD8^+^ T cells, B cells, and natural killer (NK) cells, play an important role in the fight against the virus [8]. Additionally, immune disorders are an important factor in the development of severe COVID-19 [9]. In clinical practice, there are relatively few immune indicators for clinicians, including nonspecific tests involving blood routine, lymphocyte subsets, and specific immunoglobulin *M* (IgM) and immunoglobulin *G* (IgG) in serum. Recent studies have shown that lymphopenia (<20%) and severe lymphopenia (<5%) are observed in severe cases and CD8^+^ T cells can be a predictor of severe disease [9]. Moreover, virus-specific IgM increases followed by virus-specific IgGare detected during convalescence in the acute phase [10, 11]. The major discharge criteria are the novel coronavirus negative test on nasal and pharyngeal swabs. Since many nonsevere patients are self-quarantined at home once diagnosed in most countries, few studies have focused on the relationship between nucleic acid positive duration (NPD) of nonsevere novel coronavirus and immunological features in these cases. In China, all COVID-19 patients must be isolated and treated in designated hospitals. Therefore, we performed a comprehensive evaluation of the characteristics of 122 COVID-19 patients admitted to the designated hospital in Tianjin, China (Tianjin Haihe Hospital). Herein, we analyzed the clinical and immunological features of nonsevere cases. Our current findings might help in the understanding of SARS-CoV-2 nucleic acid change in these cases, and the relationship between immunological indexes and COVID-19 prognoses.

## 2. Methods

### 2.1. Patients

Patients with COVID⁃19 were recruited at the Tianjin Haihe hospital from May 2020 to June 2021. The diagnostic criteria and clinical classification were based on the National Health Commission of China [12]. This was a retrospective study and no evidence that the enrolled patients were infected with a predominance of variants of concern was detected. After discharge, patients were kept under observation and health monitoring for 14 d.

### 2.2. Date Collection

Demographic data (age, sex), epidemiological contact history, onset date, basic diseases, fever, hyposmia, blood routine (LYM), liver and kidney function, SARS-CoV-2 antibody, specific IgM and IgG, T lymphocyte subsets, and other data were collected at the hospital. The NPD was evaluated by throat or nose swabs and was defined as the time from the first positive to the first negative with a parallel controlled trial verified by the local Disease Control and Prevention Center.

### 2.3. Statistical Analyses

The data were analyzed by SPSS 25.0 statistical and GraphPad Prism 8.0 software. Data are described as means, interquartile ranges, or percentages of the relative frequency according to the difference, and the 95% confidence interval (CI) was used. The *P* values of comparisons between mild and moderate cases were derived from *χ*^2^, Fisher's exact, or unpaired two-sided Student's *t*-tests. Furthermore, we performed a correlation analysis between the NPD, antibodies, and T-cell subtypes. A *P* < 0.05 was considered statistically significant.

## 3. Results

**Figure 1:**
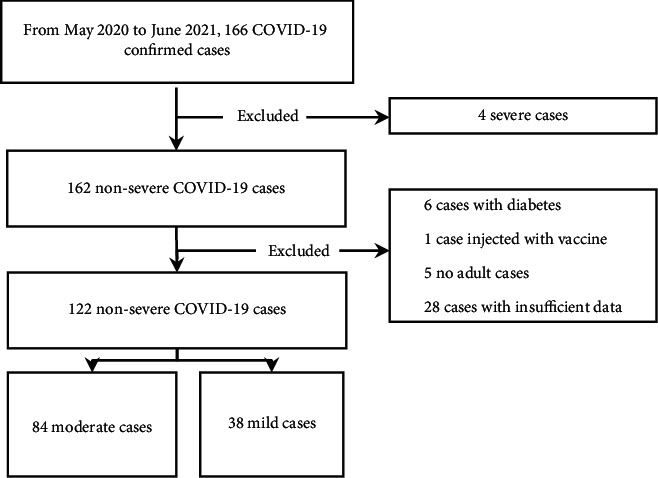
Flowchart of screening nonsevere COVID-19 confirmed cases.

From May 2020 to June 2021, 166 COVID-19 cases were confirmed. According to the guidelines for diagnosis and management of COVID-19 (8th edition, in Chinese) issued by the National Health Commission of China [12], they were divided into mild, moderate, severe, and critical severe cases. Four severe cases and no critical severe cases were detected. Hence, 162 patients were diagnosed with nonsevere COVID-19. Furthermore, six patients with diabetes, one patient that received the vaccine, five no adult cases, and 28 cases with insufficient data were excluded. Finally, the remaining 122 cases were enrolled in the present study, including 38 mild and 84 moderate cases. The flowchart of the screening of nonsevere COVID-19 confirmed cases is presented in Figure 1.

The average age of mild patients was 32.16 years and differed from moderate patients (39.50 years; *P* < 0.001). Six patients were Europeans and Americans and 116 were Asians. Fifteen patients were smokers. Fever was the most common symptom with a proportion of 23.77%. Eight patients complained of hyposmia and it was more frequent in mild cases (*P* < 0.001). The NPD was 20.49 (17.50–3.49) days in all nonsevere COVID-19 cases, and the two groups did not differ. The demographics and baseline characteristics of nonsevere COVID-19 patients are shown in Table 1.

The laboratory tests of patients presented LYM of 1.94 (CI 1.76–2.11) × 10^9^/L, alanine aminotransferase (ALT) of 29.10 (CI 26.39–31.80) U/L, and Albumin (ALB) of 45.51 (CI 39.27–51.75) g/L. The specific IgM and IgG for COVID-19 were detected by chemiluminescence. The lymphocyte subsets in the blood were analyzed by flow cytometry and the normal range of CD4^+^T cell and the CD8^+^T cell counts were 561 to 1137/MCL and 404 to 754/MCL, respectively. The levels of specific IgM and IgG for COVID-19 were higher in mild cases compared to moderate cases (*P*=0.023 and *P*=0.047, respectively). The counts of CD4^+^T and CD8^+^T cells were in the normal range in most cases, but the count of mild patients was higher than moderate ones. The immunological indexes of male and female nonsevere COVID-19 patients did not differ (Table 2).

**Figure 2:**
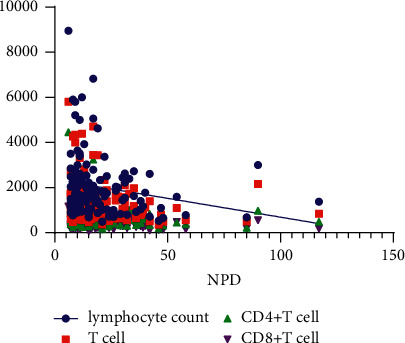
The NPD of COVID-19 has a linear relationship with lymphocyte count and T-cell subtype. NPD, nucleic acid positive duration.

The correlation analysis for specific antibodies and T cell subtypes showed that LYM, T cells, CD4^+^T cells, and CD8^+^T cells had a linear correlation with the NPD (Table 3, Figure 2). Additionally, 93 patients were monitored during their treatments and 62 COVID-19 cases presented a progressive rising trend of IgM and IgG levels. The daily increase rate of IgM and IgG was 38.42% (CI 28.22–48.61%) and 24.90% (CI 0.23–29.58%), respectively. The analysis of T-cell subtypes from 20 nonsevere COVID-19 patients showed that the count of T cells, CD4^+^T cells, and CD8^+^T cells did not differ between admission and discharge (Table 4).

## 4. Discussion

The COVID-19 infectious cases can be divided into mild, moderate, severe, and critical severe cases according to the guidelines for diagnosis and management of COVID-19 (8th edition, in Chinese) issued by the National Health Commission of China [12]. Mild and moderate cases are considered nonsevere with respiratory symptoms of fever, imaging manifestations of pneumonia, no progressive dyspnea, and oxygenation index greater than 300. At the early stage of the COVID-19 pandemic, the investigation of 132 patients from the Wuhan Fourth Hospital who had COVID-19 from February 1 to 29 showed that the proportion of nonsevere cases was over 68.19% [13]. Herein, four cases were evaluated as severe or critical cases, and the remaining 162 (97.60%) were mild or moderate cases. The reason for this phenomenon might be that the patients admitted were imported from other countries and most of them were healthy adults in the past.

Different factors can affect the human immune status. The immune system becomes mature at about 20 years in humans, and the immune system function progressively declines after 60 years [14]. Some underlying diseases, such as diabetes, can also affect the function of the immune system. Additionally, the metabolic alterations of patients suffering from obesity and diabetes mellitus (DM) can further affect the differentiation, function, and survival of components of the innate and adaptive immunities [15, 16]. COVID-19 patients with DM or obesity are at higher risk of death and are characterized by a state of chronic and low-grade inflammation because of the impaired innate and adaptive immune responses [17]. COVID-19 candidate vaccines induce a highly potent SARS-CoV-2 neutralizing antibody response [18] and elicit human antibody and TH1 T cell responses [19]. Therefore, we excluded six patients with diabetes, one patient that received the vaccine, five patients under 18, and 28 cases with inefficient data. Finally, a total of 122 patients were enrolled in the present study, the age range was 20–59 years, and included 38 mild and 84 moderate patients.

In these cases, mild COVID-19 patients were younger than moderate ones (*P* < 0.001). Cough and fever were the most common respiratory symptoms, but hyposmia was detected in 23.68% of mild and 1.09% of moderate cases (*P* < 0.001). The LYM and CD8^+^T cell counts were statistically different between the two groups, consistent with previous studies [20, 21]. Similar to many other viral infections, an increase in specific IgM in the acute phase followed by an increase in specific IgG at later phases has been observed in the course of COVID-19 [14]. An early increase in IgM followed by the development of IgG is a normal expected antibody response [9]. In the present study, the daily increase rates of IgM and IgG were also calculated using data from dynamic antibodies from 62 patients. The daily increase rates of IgM and IgG were 38.42% (CI 28.22–48.61%) and 24.90% (CI 0.23–29.58%), respectively. The correlation analysis showed that the NPD was not related to the levels of specific IgM and IgG at admission and their daily increase rates. Thus, the levels of specific antibodies against the virus varied between cases. Additionally, we focused on the NPD of nonsevere COVID-19, which was 20.49 (CI 17.50–3.49) days in the whole group and did not differ between the two groups. The correlation analysis showed that LYM, T cells, CD4^+^T cells, and CD8^+^T cells presented a linear correlation with NPD. Meanwhile, the dynamic T-cell subtypes presented an increasing trend in 20 COVID-19 cases. Hence, we demonstrated that LYM and T cell subtypes were dominant in the response of the new coronavirus.

However, our study also has some limitations. First, we did not observe the dynamic changes of specific antibodies and T-cell subtypes. Second, the postdischarge immune status of COVID-19 patients was not further assessed in the follow-up. Third, some immunological indicators, including cytokines, were not observed due to limited conditions.

In summary, the identification of specific IgM and IgG for COVID-19 is necessary, but their levels and daily increase rates might vary between cases. Moreover, the NPD presented a linear correlation with LYM, T cells, CD4^+^ T cells, and CD8^+^ T cells. Hence, more attention should be paid to these indexes in the clinic. Our current findings provided a deeper insight into the disease's pathogenesis and might help in the precise therapeutic strategy for each COVID-19 patient.

## Figures and Tables

**Table 1:** Patient demographics and baseline characteristics of nonsevere COVID-19.

	All cases (*n* = 122), mean (CI 95%)	Mild cases (*n* = 38), mean (CI 95%)	Moderate cases (*n* = 84), mean (CI 95%)	*P* value
Female, *n* (%)	40 (32.79%)	10 (26.32%)	30 (35.71%)	0.306
Age, median years (years)	37.21 (35.28–39.15)	32.16 (29.09–35.23)	39.50 (37.17–41.83)	<0.001
BMI value (kg/m^2^)	24.75 (24.05–25.45)	24.53 (23.02–26.04)	24.85 (24.07–25.62)	0.706
Smoker, *n* (%)	15 (12.30%)	7 (18.42%)	8 (9.52%)	0.232
Dyspepsia, *n* (%)	3 (2.46%)	1 (2.63%)	2 (2.38%)	1.000
Cough, *n* (%)	24 (19.67%)	9 (23.68%)	15 (17.86%)	0.453
Fever, *n* (%)	29 (23.77%)	12 (31.58%)	17 (20.24%)	0.173
Hyposmia, *n* (%)	10 (8.20%)	9 (23.68%)	1 (1.09%)	<0.001
Blood's LYM, *n* ×*n* 10^9^/L	1.94 (1.76–2.11)	2.42 (1.97–2.88)	1.72 (1.59–1.85)	0.004
ALT in serum (U/l)	36.38 (31.08–41.68)	35.17 (23.30–47.05)	36.92 (31.21–42.63)	0.764
AST in serum (U/l)	29.10 (26.39–31.80)	31.08 (24.00–38.16)	28.20 (25.80–30.61)	0.441
ALB in serum (g/l)	45.51 (39.27–51.75)	54.53 (34.37–74.69)	41.43 (40.12–42.74)	0.197
IgM of COVID-19	17.20 (9.46–24.94)	7.41 (1.76–13.07)	21.63 (10.72–32.54)	0.023
IgG of COVID-19	24.42 (17.10–31.75)	15.13 (5.79–24.47)	28.63 (18.88–38.38)	0.047
CD4^+^T cell count (×10^6^/L)	814.69 (704.69–924.68)	943.23 (753.01–1133.45)	756.54 (621.23–891.85)	0.120
CD8^+^T cell count (×10^6^/L)	483.89 (423.31–544.46)	606.30 (477.12–735.47)	428.51 (364.25–492.77)	0.007
NPD, days	20.49 (17.50–3.49)	24.53 (14.32–28.21)	24.8 5 (17.01–23.27)	0.733

BMI, body mass index; LYM, absolute value of lymphocyte count; ALT, alanine aminotransferase; AST, aspartate aminotransferase; ALB, albumin; n, number; IgM, specific immunoglobulin *M* (IgM); IgG, specific immunoglobulin G; NPD, nucleic acid positive duration.

**Table 2:** Comparison of immunological tests in male and female nonsevere COVID-19 patients.

	Male, mean (CI 95%)	Female, mean (CI 95%)	*P* value
Blood's LYM (×10^9^/L)	2.03 (1.81–2.24)	1.75 (1.44–2.05)	0.130
IgM of COVID-19	18.41 (41.64–44.28)	14.72 (3.60–25.85)	0.660
IgG of COVID-19	22.77 (13.30–32.24)	27.81 (16.25–39.38)	0.500
CD4^+^T cell count (×10^6^/L)	803.46 (690.67–916.25)	837.71 (585.81–1089.615)	0.803
CD8^+^T cell count (×10^6^/L)	506.46 (429.31–83.62	437.60 (339.05–536.16)	0.293

LYM, absolute value of lymphocyte count; IgM, specific immunoglobulin *M* (IgM); IgG, specific immunoglobulin *G*.

**Table 3:** Correlations analysis involved antibody and T-cell subtype.

		Age	BMI	IgM	IgG	LYM	T cCell	CD4^+^ T cell	CD8^+^ T cell
NPD	r	0.03	0.14	−0.04	−0.10	−0.20	−0.20	−0.23	−0.20
	*P* value	0.780	0.132	0.627	0.285	0.025	0.026	0.011	0.028

NPD, nucleic acid positive duration; BMI, body mass index; LYM, absolute value of lymphocyte count; IgM, specific immunoglobulin *M* (IgM); IgG, specific immunoglobulin *G*.

**Table 4:** The changes of T-cell subtype of COVID-19.

	At admission (*n* = 20), mean (CI 95%)	On discharge (*n* = 20), mean (CI 95%)	*P* value
LYM, (×10^6^/L)	1794.70 (1134.23–2455.17)	2181.20 (172.35–2590.04)	0.304
T-cell count (×10^6^/L)	1202.03 (751.48–1652.57)	1491.09 (1228.91–1753.26)	0.253
CD4^+^ T-cell count (×10^6^/L)	693.30 (388.74–997.86)	830.69 (675.80–985.59)	0.405
CD8^+^ T-cell count (×10^6^/L)	393.09 (256.79–529.39)	538.24 (431.90–644.58)	0.087

LYM, absolute value of lymphocyte count;. n, number.

## Data Availability

The datasets used and analyzed during this study are available from the corresponding author upon request.
